# Grating-based spectral X-ray dark-field imaging for correlation with structural size measures

**DOI:** 10.1038/s41598-020-70011-3

**Published:** 2020-08-06

**Authors:** Kirsten Taphorn, Fabio De Marco, Jana Andrejewski, Thorsten Sellerer, Franz Pfeiffer, Julia Herzen

**Affiliations:** 1 Chair of Biomedical Physics, Department of Physics and Munich School of BioEngineering, Technical University of Munich, James-Franck-Straße 1, 85748 Garching, Germany; 2 Department of Diagnostic and Interventional Radiology, Klinikum rechts der Isar, Technical University of Munich, Ismaninger Straße 22, 81675 München, Germany; 3 Institute for Advanced Study, Technical University of Munich, 85748 Garching, Germany

**Keywords:** Radiography, Imaging techniques

## Abstract

X-ray dark-field (XDF) imaging accesses information on the small-angle scattering properties of the sample. With grating interferometry, the measured scattering signal is related to the sample’s autocorrelation function, which was previously demonstrated for simple samples, such as mono-dispersed microspheres for which the autocorrelation function is mathematically given. However, in potential clinical applications of XDF imaging, complex microstructures, such as lung parenchyma are under investigation. Their bahaviour in XDF imaging is not yet known and no mathematical description of the autocorrelation function is derived so far. In this work we demonstrate the previously established correlation of the XDF data of complex sample structures with their autocorrelation function to be impractical. Furthermore, we propose an applicable correlation between XDF and the sample’s structural parameter on the basis of mean chord length, a medically-approved measure for alveolar structure, known to be affected by structural lung diseases. Our findings reveal a correlation between energy-dependent XDF imaging and the sample’s mean chord length. By that, a connection between a medical measure for alveoli and XDF is achieved, which is particularly important regarding potential future XDF lung imaging applications for the assessment of alveoli size in diagnostic lung imaging.

## Introduction

The main application of X-rays is in diagnostic imaging, which is based on their ability to penetrate matter and show the body’s internal structure noninvasively. Various modalities, such as radiography, computed tomography (CT) and mammography are based on the interaction of X-rays with matter and are highly developed. Up to now, image formation in clinical state-of-the-art X-ray applications is based on attenuation. In recent years, several X-ray imaging techniques, such as analyzer-based imaging^1,2^, grating-based imaging^3^ and edge illumination modalities^4^, were developed. They are able to access information not only about the attenuation properties of the observed object, but also of phase and scattering properties. Thus, information about the sample’s microstructure and refractive properties, related to ultra-small-angle X-ray scattering, can be accessed^5,6^. Furthermore, previous studies have demonstrated a relation between the scattering signal and the size of structural features for the prior mentioned imaging modalities^7,8^.

In terms of clinical applicability, grating-based X-ray dark-field (XDF) imaging with a three grating Talbot-Lau interferometer shows promise as it is feasible with conventional X-ray sources and does not require high spatial or temporal coherence^9^. In recent years, previous studies aimed to provide a quantitative correlation between XDF signal at a distinct sampled correlation length and the autocorrelation function of the sample’s microstructure. It was derived theoretically and subsequently shown experimentally by Lynch et al.^10^ and Yashiro et al.^11^ with monochromatic X-ray sources. Further, Prade et al.^8^ extented the experimental proof of the correlation to a polychromatic laboratory grating-based XDF setup by reference to an adequately simple problem formulation with mono-dispersed microspheres.

As XDF imaging is highly depending on the structure of the sample on the micometer scale, diagnostic lung imaging is a highly promising application of grating-based XDF imaging. The air-tissue interfaces formed by alveolar walls cause small-angle X-ray scattering and thus a strong XDF signal, especially in comparison to the surrounding soft tissue in the thorax^12^. Among the most important structural lung pathologies are pulmonary emphysema and fibrosis, which differ in microstructure. Emphysema presents as a destruction of alveolar structure, whereas fibrosis is linked to the formation of excess tissue and densification of alveoli^13^. A widely used quantity for the characterization of the lung parenchyma is the mean chrod length (MCL). It is a mean intercept measure, well-established in histological analysis of lung tissue and was first introduced by Campbell and Tomkeieff^14^, based on calculations for convex shapes presented by Tomkeieff^15^. The MCL describes the entire acinar airspace complex^16^ and is known to be affected by several structural lung diseases. On the one hand, emphysema leads to an increased MCL^17^, on the other hand, fibrotic lung tissue is demonstrated to show smaller MCLs compared to healthy lung tissue^18^.

The diagnostic potential of XDF iamging is given due to the correlation of structure size and XDF signal. Thus, the difference in microstructure of the lung pathologies results in a variation of XDF signal. Recent small-animal studies revealed a decrease in XDF signal for both emphysema^19^ and fibrosis^20^, which suggests a similar behaviour for humans and would enable a diagnosis of potential pathological changes.

However, the quantitative correlation between XDF and the autocorrelation function was only demonstrated for spheres. But lung parenchyma can not be approximated as sphere-like structures, but rather are a complex system of alveoli walls and air-filled spaces. The purpose of the present work is to demonstrate the difficulty to relate the autocorrelation function and XDF imaging of complex sample structures quantitatively. Furthermore, we present an alternative correlation between energy dependent XDF imaging and the sample’s MCL, which is shown to be more practicable for fututre XDF imaging applications on samples with complex microstructure, such as lung parenchyma.

## Methods

In grating-based XDF imaging, three contrast channels are retrieved, namely transmission, differential phase and dark-field. With the phase-stepping approach^21^, an intensity curve is acquired for each pixel. The visibility of this curve is defined by its maximum and minimum intensity. Based on the visibility acquired with and without the sample, the dark-field signal *D* can be calculated as the reduction of visibility due to scattering^3^.

The length scale on which correlations within the sample can be measured in grating-based XDF imaging is given by the autocorrelation length $\xi _\text{corr}$. It depends on the wavelength $\lambda$ of the X-ray beam as well as the placement of the sample inside the grating interferometer $d_{\text{S,G}_{2}}$ given by the distance between the sample, and the analyzer grating and the grating period $p_{\text{G}_{2}}$ of the analyzer grating^10^,

1 $$\xi _{{{\text{corr}}}} (E) = \frac{{d_{{S,G_{2} }} }}{{p_{{G_{2} }} }} \cdot \lambda = \frac{{d_{{S,G_{2} }} }}{{p_{{G_{2} }} }} \cdot \frac{{hc}}{E}.$$

With a grating interferometer, the differential scattering cross-section is transformed back into the real-space. Thus, in the case of isotropic multiple scattering, the XDF signal can be directly related to the autocorrelation length $\xi _\text {corr}$ via the projected real-space correlation function $G(\xi _\text {corr})$ of the sample and its thickness *t*^8,11,22^,

2 $$\begin{aligned} -\text {ln}[D(\xi _\text {corr})] = \sigma t[1-G(\xi _\text {corr})]. \end{aligned}$$

The scattering cross-section $\sigma$ is proportional to $\lambda ^2$. As both $\sigma$ and $\xi _{\text {corr}}$ are depending on the X-ray wavelength, the dark-field signal is a function of the X-ray energy. The projected real-space correlation function of the sample’s electron density is defined as^23^,

3 $$\begin{aligned} G(\xi _\text {corr})=\frac{\int \gamma (x,y=0,z=\xi _\text {corr}) dx}{\int \gamma (x,y=0,z=0) dx}. \end{aligned}$$

In terms of XDF imaging, *x* denotes the beam direction. The *z*-direction corresponds to the sensitivity direction of the setup, which is perpendicular to the grating lamellae as well as *x*. The autocorrelation function $G(\xi _\text {corr})$ is an unit-less function with $G(0)=1$ and converges to 0 for $\xi _\text {corr} \rightarrow \infty$ for samples without long-range ordering. Mathematically, the autocorrelation of a function gives the correlation of the function with a shifted copy of itself and consequently, is a function of the shift. According to the correlation theorem^24^, the normalized autocorrelation function $\gamma (\mathbf {r})$ of the sample’s electron density $\rho (\mathbf {r})$ in the considered volume is given by,

4 $$\begin{aligned} \gamma {(\mathbf {r})}=\frac{\int _V \Delta \rho (\mathbf {r'})\Delta \rho (\mathbf {r'}+\mathbf {r}) d\mathbf {r'}}{\int _V \Delta \rho (\mathbf {r'})\Delta \rho (\mathbf {r'}) d\mathbf {r'}}= F^{-1} [ |F(\Delta \rho (\mathbf {r}))|^2 ]=F^{-1}[F(\Delta \rho (\mathbf {r}))\cdot F^*(\Delta \rho (\mathbf {r}))], \end{aligned}$$

where $\Delta \rho (\mathbf {r})$ is given by subtraction of its mean value $\Delta \rho (\mathbf {r})=\rho (\mathbf {r})-\langle \rho (\mathbf {r})\rangle$^23^. *F* denotes the three-dimensional Fourier transform and its inverse is termed $F^{-1}$. The last equality uses $|F(x)|^2=F(x)\cdot F^*(x)$.

Equation (2) was proven by Prade et al.^8^ on the example of mono-dispersed microspheres and the use of the mathematical discription of $G(\xi _\text {corr})$ for spheres. The term $[1-G(\xi _\text {corr})]$ increases with increasing $\xi _\text {corr}$, in the relevant range for grating-based XDF imaging. Thus, $-\text {ln}[D(\xi _\text {corr})]$ increases with $\xi _\text {corr}$ and decreases with *E* (due to inverse proportionality in equation (1)). When considering spheres with a radius smaller compared to the sample correlation length, the energy dependency of $-\ln (D)$ is given by the scattering cross section with $E^{-2}$. If the radius is larger compared to the sampled correlation length, the energy dependency can vary up to $E^{-4}$. Therefore, samples with different sizes of microstructure have different energy-dependencies in XDF imaging.

For non-spherical structures, the autocorrelation function is mathematically not known. Therefore, we calculated $G(\xi _\text {corr})$ based on microCT data, which provides the spatial distribution of the attenuation coefficient $\mu (x,y,z)$^25^. Assuming that the chemical composition is constant throughout the sample, the attenuation coefficient is directly proportional to the electron density $\rho (x,y,z)$ and can be used for the calculation of $G(\xi _\text {corr})$. For a correlation between the autocorrelation function of the sample and the XDF signal sampled at distinct autocorrelation lengths, multiple autocorrelation lengths have to be measured. For that purrpose, the position of the sample within the interferometer ($d_{\text{S,G}_{2}}$ in Eq. 1) could be changed between the single measurements. But this approach has its limitations, especially when operating in cone-beam geometry. Due to alternating sample magnification, additional computational steps for image registration are required^26^. A change in the period of the analyzer grating ($p_{\text{G}_{2}}$ in Eq. 1) in between two measurements is experimentally not practicable. Therefore, in our experiments, we achieved a range of sampled autocorrelation lengths by imaging with different X-ray spectra. The source was a conventional polychromatic X-ray source, the energy discrimination was done by spectral detection. In recent years, photon-counting detectors were developed. They have the ability to measure the incoming X-ray intensities in several energy intervals simultaneously, meaning that the signal is divided into different photon spectra^27^. By that, the energy information is not lost^28^. Therefore, it is possible to image a sample with several different photon spectra within one measurement. In view of potential clinical applications, the use of spectral detection provides a time-efficient imaging method as well as potentially reduced dose, compared to spectral X-ray imaging with a dual-source systems. In our experiments we used a two-threshold photon-counting detector. Thus, within one measurement two images for two different energy intervals (low energy interval: 23–64 keV; high energy interval: 64–120 keV) were aquired.

**Figure 1:**
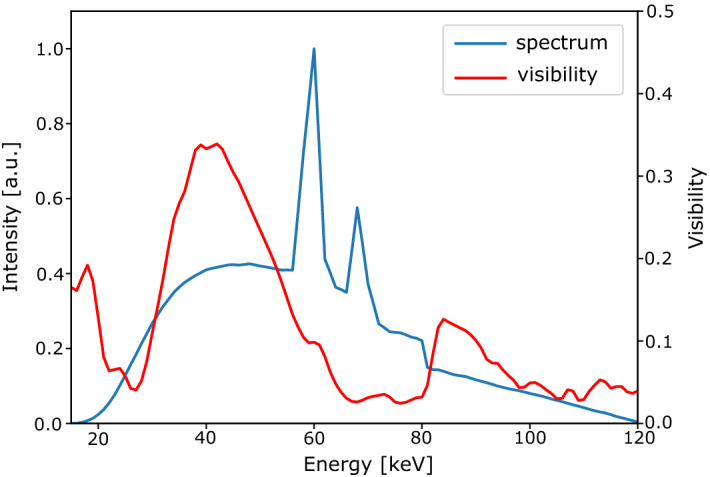
Simulated source spectrum and measured visibility spectrum. The source spectrum $\Phi (E)$ (blue) was simulated based on the experimental source parameters. The measured visibility spectrum *V*(*E*) has its maximum at the design energy of 45 keV. Due to the absorbtion edge of gold, the visibility decreases between 60 keV and 80 keV. The second visibility peak is approximately at twice the design energy.

The XDF signal measured with a polychromatic source can be interpreted as the summation over all dark-field signals for each individual sampled autocorrelation length of different photon energies. We assume that for a polychromatic X-ray beam, the effectively sampled autocorrelation length $\langle \xi _{\text {corr}}\rangle _\text {w}$ will be the mean value of sampled autocorrelation lengths for all energies from the polychromatic spectrum, whereby the contribution of each distinct energy to the autocorrelation length is depending on the visibility spectrum *V*(*E*) of the setup and the effective spectra $\Phi _\text {eff}(E)$. The effective spectra for each energy bin are given by the incident spectrum multiplied with the response function of the detector summed up over the energy range of each bin. For that, the response functions of the photon-counting detector were simulated based on the approach of Schlomka et al.^29^ and tuned via calibration measurements for the specific detector model used in our experiments. The effectively sampled autocorrelation lengths $\langle \xi _{\text {corr}}\rangle _\text {w}$ can be determined as a weighted mean value,

5 $$\begin{aligned} \langle \xi _{\text {corr}}\rangle _\text {w}=\frac{\int _{E_1}^{E_2}\Phi _\text {eff}(E) \cdot V(E)\cdot \xi _{\text {corr}}(E) dE}{\int _{E_1}^{E_2}\Phi _\text {eff}(E) \cdot V(E) dE}, \end{aligned}$$

where $\xi _{\text {corr}}(E)$ is given by Eq. (1). The contribution of each energy to the measured dark-field signal is indicated by the weighting with the effective photon and visibility spectrum. This takes into account that the contribution to *D* by photons with energies of low flux is minor, compared to photon energies of higher flux. The same applies for photon energies for which the visibility is low. The visibility spectrum gives the visibility of the XDF setup depending on the X-ray energy and is independent of the accelaration voltage of the X-ray source. Typically, the peak visibility is located near the design energy of the grating interferometer. We measured the visibility spectrum *V*(*E*) with an X-ray spectrometer by Amptek, Inc. (Bedford, Massachusetts, USA). The photon spectrum was simulated based on the applied accelaration voltage of 120 kV. Additionally, the absorption due to the gold gratings of the interferometer was taken into account. Figure 1 shows the measured visibility spectrum in red. The peak visibility is at 45 keV, the visibility drops until it reaches a minimum at 64 keV and again increases, which is due to the high transmittance of gold below the K-edge.

## Results

In this work, we investigated samples with complex micro structure, such as various types of closed-cell plastic foams, namely: neoprene (CR-L), ethylene propylene diene monomer rubber (EPDM) (W. KÖPP GmbH & Co. KG, Aachen, Germany), and polyurethane (PU) rubber (Rodgers Germany GmbH, Eschenbach, Germany). Addtionally, non-suspended hollow glass microspheres, namely K1 and S60 (3M, St. Paul, USA) were investigated.

**Figure 2:**
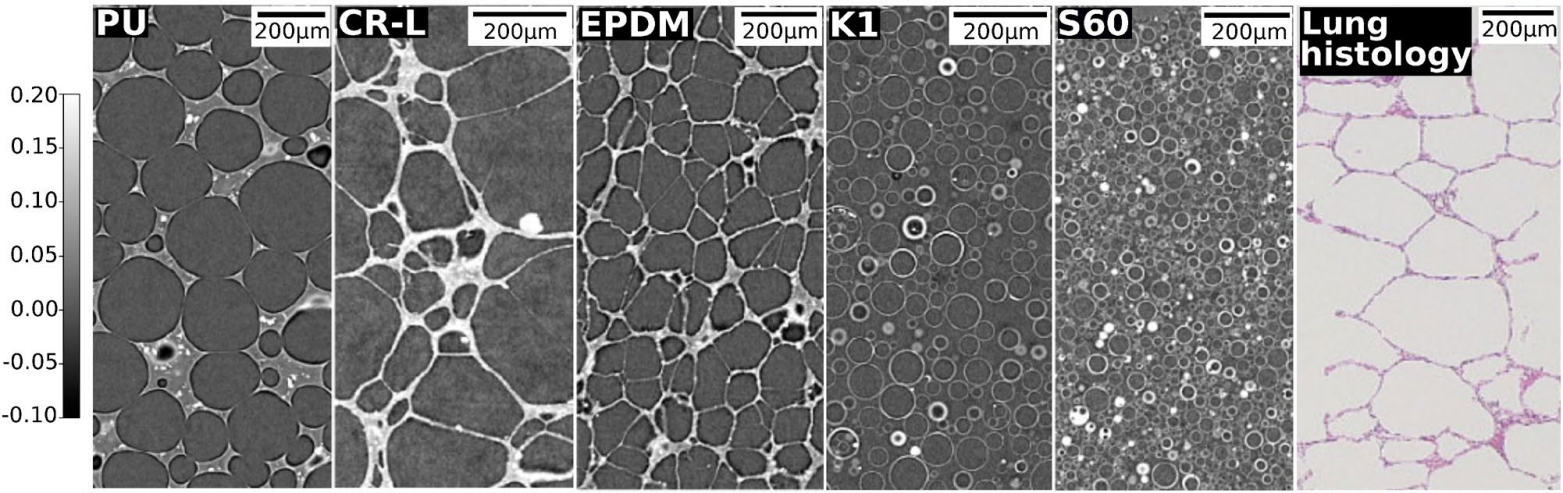
MicroCT slices of sample materials in comparison to lung histology. MicroCT slices for different types of closed-cell plastic foams (PU, CR-L and EPDM) as well as hollow glass microspheres (K1 and S60) shown in comparison to a histological slice of healthy human lung tissue (Adapted from Woods et al.^30^). Color bar applies to the microCT slices.

An example CT slice for the three different types of closed-cell foams (PU, CR-L and EPDM) as well as the two types of hollow glass microspheres (K1 and S60) are depicted in Fig. 2. Evidently, the size of their structure varies in terms of cell size and ratio of material and air. Addiditionally, the foams visually highly resemble the structure of the human lung parenchyma of which a histological slice is provided in Fig. 2.

Due to the differences in structure size among the sample materials under investigation, we expect them to appear differently in XDF imaging. In our experiments, energy-dependent XDF signals were measured at a polychromatic setup with a two-threshold photon-counting detector.

**Table 1:** Measured dark-field signals for all sample materials under investigation for both energy intervals.

	$$-\text {ln}(D)$$				
	CR-L	EPDM	PU	K1	S60
Low energy interval:	1.07	1.05	0.50	0.65	2.05
High energy interval:	0.36	0.37	0.13	0.24	0.93

For both energy intervals, the measured XDF signals for the sample materials under investigation are provided in Table 1. As expected, $-\ln (D)$ in the low energy interval is higher compared to the high energy interval. In small-angle X-ray scattering, lower X-ray energies are related to larger scattering angles. Consequently, the visibility is more strongly reduced in the low energy interval resulting in a higher XDF signal. Furthermore, the dark-field signal varies among the sample materials, as expected.

### Projected real-space correlation function

According to Eq. (1), the two energy intervals are related to different sampled autocorrelation lengths $\xi _\text {corr}$. The effectively sampled autocorrelation length was determined for each energy interval by Eq. (5). For the low energy interval, the autocorrelation length was $\langle \xi _{\text {corr}}\rangle _\text {w,l}=1.65\,\upmu$m. The corresponding X-ray energy is $E_\text {w,l}=43.0$ keV with a wavelength of $\lambda _\text {w,l}= 0.29\, \overset{\circ}{\mathrm{A}}$. Analogously, for the high energy interval the sampled autocorrelaiton length was $\langle \xi _{\text {corr}}\rangle _\text {w,h}=0.88\,\upmu$m and a weighted X-ray energy of $E_\text {w,h}=80.9$ keV with wavelength $\lambda _\text {w,h}= 0.15\,\overset{\circ}{\mathrm{A}}$ was calculated. Considering the dark-field measurements from Table 2, all sample materials provide a higher dark-field signal at smaller $\xi _\text {corr}$ and thus, follow the trend predicted by Eq. (2).

**Figure 3:**
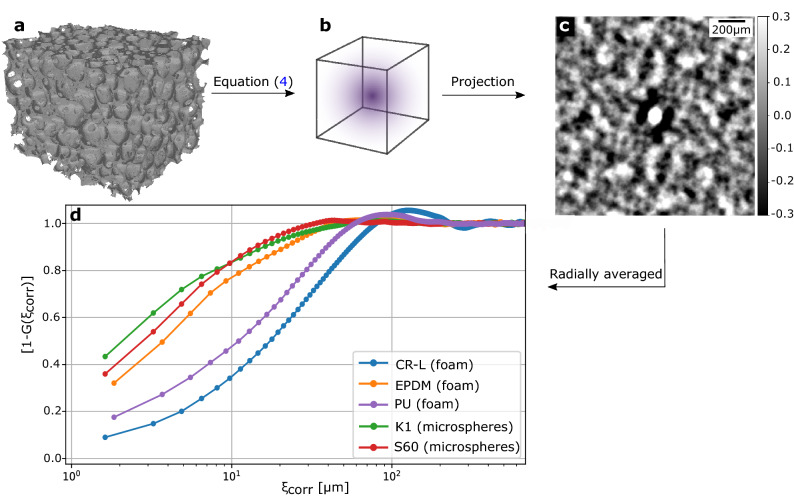
Determination of projected real-space correlation function. The three-dimensional microCT data set (**a**) is Fourier transformed and multiplied with its complex conjugate. To extract the autocorrelation function $\gamma (x,y,z)$ (sketched in **b**), an inverse Fourier transform is performed (compare Eq. (4)). After calculating the one-dimensional projection (**c**), the projected real-space correlation function $G(\xi _{\text{corr}})$ is given as the radial average of (**c**). (**d**) For several phantom materials, $[1-G(\xi _{\text{corr}})]$ is plotted as a function of the autocorrelation length $\xi_{\text{corr}}$.

In a next step, we caluclated the real-space autocorrelation function of the (mean-normalized) attenuation coefficients, from the microCT data sets. Figure 3 illustrates the process on the example of PU. In Fig. 3a, $\Delta \mu (x,y,z)$ in a region of interest is depicted. By applying Eq. (4), the autocorrelation function $\gamma [\Delta \mu (x,y,z)]$ is obtained and the peak at $(x,y,z)=(0,0,0)$ is normalized to 1 (compare normalization in Eq. (3)). After an one-dimensional projection of $\gamma [\Delta \mu (x,y,z)]$ (Fig. 3b), the projected autocorrelation function [*G*(*x*, *z*) in Fig. 3c] is obtained. As grating-based XDF imaging is only sensitive to scattering perpendicular to the beam and grating lamellae, theoretically only single points along one distinct radial profile of *G*(*x*, *z*) are measured. Due to assumed isotropy of the samples under investigation, better statistics is obtained by averaging over all angular radial profiles, which yields the unit-less projected real-space autocorrelation function *G* depending on $\xi _\text {corr}$. Figure 3d depicts $[1-G(\xi _\text {corr})]$ depending on the autocorrelation length $\xi _\text {corr}$ for all sample materials used in the experiments. It can be seen that the initial slopes and maxima are different for each type of sample material.

For increasing $\xi _{\text {corr}}$ a decrease of the autocorrelation function $G(\xi _{\text {corr}})$ is expected. Thus, the increasing functions $[1-G(\xi _{\text {corr}})]$ for the different sample materials follow the predicted trend. The resolution of the projected real-space correlation function is limited by the resolution of the microCT data sets as the minimal possible shift for the calculation of the autocorrelation function $\gamma [\Delta \mu (x,y,z)]$ is one voxel. The reason for $G(\xi _\text {corr})$ attaining negative values is the subtraction of the mean value prior to Eq. (4). The location of the maximum of $[1-G(\xi _\text {corr})]$ is referred to a shift of the volume, at which the similarity is minimal. In case of spheres, this can be referred to the radius.

Based on Eq. (2) and the energy dependency of the scattering cross section of $E^{-2}$, we divided the negative logarithmic dark-field signals $-\text {ln}[D(\xi _\text {corr})]$ by the squared wavelengths $\lambda ^2_\text {w,l}$ and $\lambda ^2_\text {w,h}$ of the X-ray beam for low and high energy interval, respectively. The results should correlate with $[1-G(\xi _\text {corr})]$ calculated from the microCT data sets. The values of $-\text {ln}[D(\langle \xi _{\text {corr}}\rangle _\text {w})]/\lambda ^2_\text {w}$ are given in Table 2, depending on the previously calculated weighted autocorrelation length $\langle \xi _{\text {corr}}\rangle _\text {w}$.

**Table 2:** $-\text {ln}[D(\langle \xi _{\text{corr}}\rangle _\text{w})]/\lambda ^2_\text{w}$ for high ($\langle \xi _{\text{corr}}\rangle _\text{w,h}=0.88\,\upmu \text{m}$) and low ($\langle \xi _{\text{corr}}\rangle _\text{w,l}=1.65\,\upmu \text{m}$) energy interval for different sample materials

Autocorrelation length $\langle \xi _\text {corr}\rangle _\text {w}$ [$\upmu \text {m}$]	$-\ln [D(\langle \xi _{\text {corr}}\rangle _\text {w})]/\lambda ^2_\text {w}$ [$1/\overset{\circ}{\mathrm{A}}^2$]				
	CR-L	EPDM	PU	K1	S60
0.88	16.00	16.00	5.78	10.67	41.33
1.65	12.72	12.60	5.95	7.73	24.38

Although the trend in both the XDF data provided in Table 1 and the autocorrelation function from the microCT data plotted in Fig. 3 are following the expected trend, the quantitative division by $\lambda ^2_\text {w}$ results in $-\ln [D(\langle \xi _{\text {corr}}\rangle _\text {w})]/\lambda ^2_\text {w}$ values, which contrary to expectations are decreasing for increasing autocorrelation lengths. Although the values for $-\text {ln}[D(\langle \xi _{\text {corr}}\rangle _\text {w})]/\lambda ^2_\text {w}$ of PU are following the same trend compared to $[1-G(\xi _\text {corr})]$, a quantitative correlation is impracticle.

### Mean chord length

**Figure 4:**
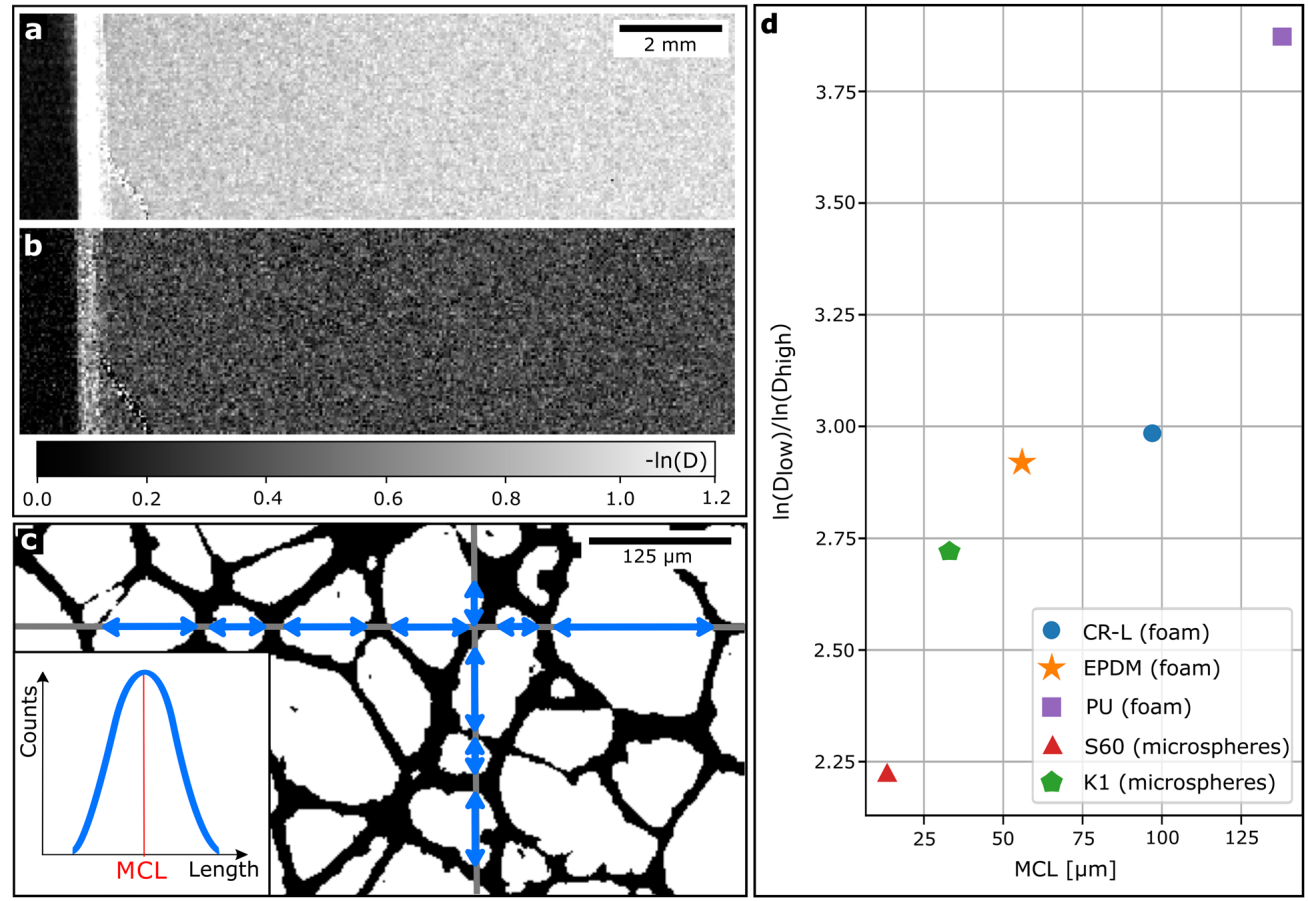
X-ray dark-field images of EPDM for low ($23{-}64$ keV, **a**) and high ($64{-}120$ keV, **b**) energy interval. (**c**) Segmented microCT slice of EPDM with example test lines (gray) for measuring the chord lengths (blue), resulting in a distribution with its mean chord length. (**d**) Obtained correlation between quotient of X-ray dark-field signals of both energy intervals and the determined mean chord lengths.

As the autocorrelation function was demonstrated to be unsuitable for a correlation between the sample’s structure size and XDF imaging, especially in view of large scale clinical applications, we demonstrate a correlation between XDF and an alternative structural parameter, namely MCL. The measurement procedure of MCL can be easily transferred from two-dimensional histological sections to two-dimensional sectional images from microCT. For that purpose, the microCT scans were binary segmented using the interactive learning and segmentation toolkit ILASTIK^31^. An example slice of EPDM after segmentation is depicted in Fig. 4c. For the determination of MCL, a sufficient number of random test lines (gray) is drawn through the sectional images of a three-dimensional region of interest. The lengths of cavities (blue) are measured along each test line and added to a distribution of chord lengths (see inset in Fig 4c), from which the material’s MCL (red) can be extracted.

If a cavity extends beyond the boundaries of the observed region of interest, it is excluded, as it would cause an underestimation of MCL. This method relies on the use of test lines, thus, isotropy is required, which can be given by the sample itself. Moreover, by randomizing the spatial orientation of the line set, isotropy can be achieved^16^. Both was constituted in our case. The MCL was measured for all sample materials under investigation.

As we expect differences in energy-dependency of the XDF signal among the sample materials, the ratio of both energy bins represents the differences qualitatively and is used for a correlation with the MCL. Dividing both energy bin images is possible as they are spatially registered by the photon-counting detector. Therefore, a finite volume element within the sample is represented in the identical pixel in both energy bins. In Fig. 4 the dark-field images of low (a) and high energy bin (b) are provided for the example of EPDM. The ratio of both energy intervals for all sample materials are depicted in Fig. 4d, depending on the measured MCL. For increasing MCL, the quotient of spectral XDF signals is increasing. This suggests that the energy-dependency of the different sample materials increases with increasing structure size and the difference in microstructure can be qualitatively depicted in spectral XDF imaging with a photon-counting detector. This correlation reveals the possibility to grade objects based on varying structural sizes in a single XDF phase-stepping with a spectral detector.

## Discussion

Although the trend of measured XDF signals depending on $\xi _\text {corr}$ follow the theoretical description, a quantitative correlation of XDF imaging with a polychromatic source and the real-space autocorrelation function for complex sample materials was demonstrated to be impracticle. Firstly, the autocorrelation function of non-spherical samples is mathematically not described and the experimental determination of the autocorrelation function in microCT is difficult especially regarding resolution at small autocorrelation lengths. Alternatively, a sampling of larger autocorrelation lengths in XDF imaging could be done, but for that the grating periods would have to be reduced according to Eq. (1), which leads to issues at fabrication. The distance between the sample and the analyzer grating $d_{S,G}$ could be increased, however, it is limited by half the maximum size of the grating interferometer. The overall length and Talbot order of the Talbot–Lau interferometer could be increased to enable larger sample to grating distances. But for potential XDF applications, the total length of the imaging setup is usually tried to be kept as small as possible. Furthermore, the information on the energy-dependency of the XDF signal is provided in our measurements by a spectral detector in combination with a polychromatic source. By that, a continuous distribution of wavelengths is used for imaging in each energy bin, which results in a superposition of multiple sampled autocorrelation lengths. The dark-field signal per pixel is consequently the weighted summation over all energies. However, for a quantitative investigation of the mathematically given correlation, the determination of an effectively sampled autocorrelation length $\langle \xi _{\text {corr}}\rangle _\text {w}$ is inevitable. Prior to the calculation of the autocorrelation length, many assumptions have to be made, regarding the source spectrum and the energy response of the detector. It has already been demonstrated that the photon-counting detector XCounter Flite X1 features a wide response function to a narrow spectrum^32^, resulting in an overlap of the spectra of both intervals, which may be higher than presumed. As a consequence, the effectively sampled autocorrelation lengths lie more closely together than calculated. To minimize uncertainties induced by the detector, an integrated charge-sharing correction was used. But also other effects, such as fluorescence of the sensor material, can cause an imprecise bin separation. No further corrections for such effects were implemented in this work. Apart from that, the determination of an effective autocorrelation length for each energy bin neglects the extension of the sample in beam direction. The correlation length decreases along the beam direction thoughout the sample. Consequently, the larger the sample thickness, the larger the range of sampled correlation lengths gets. In view of potential applications of correlations between XDF and microstructure size, this is a disadvantage. In diagnostic imaging for instance, the lung diameter of an average person is 15 cm, for which a difference in autocorrelation length from one to the other patient side is expected. Another important point is the limited number of data points given by the two avaiable energy bins which is disadvantageuos for a correlation. Hence, a higher number of smaller energy intervals could lead to more precise results by providing additional sampled correlation lengths. This could be achieved by using a photon-counting detector with more adjustable thresholds. However, photon-counting detectors with a higher number of thresholds are rarley available and a higher number of thresholds is currently not available in combination with a charge-sharing correction. The overlap of the effective spectra without charge-sharing correction will be increased and the difference in effectively sampled autcorrelation lengths decreased. Furthermore, the problems regarding the detector response and overlapping energy intervals still remain and might be enhanced.

The overall difficulty of relating the autocorrelation function and XDF signal for samples with complex and non-spherical microstructure makes its appliation impracticle. Therefore, we have demonstrated an alternative approach to correlate spectral XDF imaging with the sample’s microstructure size on the basis of MCL. Due to a difference in energy-dependent scattering properties of the sample materials. The MCL is an established medical quantity, which is known for common pathologies in the lung. In addition, the determination of MCL is robust and can be done equally for many types of porous materials. Furthermore, the MCLs of the samples under investigation were distributed over a broad range between $22~\upmu$m and $138~\upmu$m. This is slightly smaller but in the same order of magnitude compared to the MCL of human lung tissue, ranges from approximately $100~\upmu$m (fibrotic lung tissue)^18^ up to $620~\upmu$m (severe pulmonary emphysema). Healthy human lung tissue has a MCL of $220~\upmu$m^30^. As our method provides a qualitative but not quantitative correlation, no assumptions of the detector’s energy response, the source spectrum, nor the visibility spectrum have to be made. However, the choice of the threshold settings is expected to influence the results quantitatively and should preferrably be done by considering the source and visibility spectrum. The approach of accessing the sample’s structure based on the MCL with spectral XDF imaging can be performed at any grating-based interferometric setup. To enable a qualitative ranging of structure sizes based on XDF imaging, a calibration with known sample structures, for instance the sample materials used in this work, would have to be performed.

Conclusively, our findings suggest the possibility to grade objects with various structural sizes by accessing information about their structural properties in energy-dependent grating-based XDF imaging with a spectral detector. Furthermore, the demonstrated correlation between XDF imaging and a medical measure for alveolar structure implies a potential increase of diagnostic power of XDF lung imaging. With our results an attempt to directly distinguish samples with structural properties comparable to human lung tissue in spectral grating-based XDF imaging was presented. Hence, information about destruction or densification of lung tissue may be directly accessible and precise statements about structural changes in lung tissue could be made. For patients with lung diseases in early stages, XDF chest imaging could consequently allow a differentiation between pathological structural changes. In comparison to state-of-the-art chest imaging modalities, such as conventional chest radiography which typically lacks sensitivity in terms of diagnostic lung imaging, our method has the potential to increase the sensitivity in XDF applications. To further investigate the applicability of the demonstrated correlation between MCL and spectral grating-based XDF imaging, research should focus especially on the impact of additional absorbers which can cause beam-hardening and therefore falsify the measured XDF signal.

### Data acquisition

The spectral XDF measurements were performed at a polychromatic cone-beam microfocus setup. The source was the XWT-160-CT (X.ray WorX, Garbsen, Germany) X-ray tube with a tungsten reflexion target. The tube window consisted of 2 mm aluminum. The source was operated at 120 kV and 50 W. The Talbot-Lau grating interferometer consisted of three gratings and was arranged horizontally in symmetric geometry. The distance between the source and reference grating as well as the distance between reference and analyzer grating were 0.925 m, which corresponds to the first Talbot order. The total length of the grating interferometer was 1.85 m. The periods of source $\text{G}_{\rm 0}$ and analyzer grating $\text{G}_{2}$ were $p_{\text {G}_{0}}=p_{\text{G}_{2}}=10\,\upmu \text {m}$. Both consisted of gold. The reference grating $\text {G}_{1}$ was a $\pi /2$ phase-shifting grating with a period of $5\,\upmu \text {m}$. The distance between sample and analyzer grating was $d_{\text{S,G}_{2}}=0.571$ m.

The photon-counting detector XCounter Flite X1 was used, which consists of a 0.75 mm CdTe layer and its pixel size is $0.1\times 0.1$ mm$^2$. It allows for two energy thresholds to be set. By that, two dark-field images for different photon spectra are gained within a single acquisition. Energy intervals were set at the detector by consideration of the source’s photon $\Phi (E)$ and visibility spectrum *V*(*E*).

Data acquisition was performed with a phase-stepping approach^21^ with seven phase steps. For each phase step, 20 frames with $1\,\text {s}$ exposure time were acquired. Dark-field signals *D* were obtained from the raw phase-stepping by an expectation maximization algorithm as described by Marschner et al.^33^.

To access information about the structure of sample materials, microCT datasets were acquired with the ZEISS Xradia VersaXRM-500 (Zeiss, Oberkochen, Germany), a microCT system developed for non-destructive industrial testing. The tube was operated with 45 kV and 4 W. An objective with a 4$\times$ magnification was used and the voxel size was $1.62 \times 1.62 \times 1.62 ~\upmu \hbox {m}^3$ for CR-L, K1 and S60 and $1.84 \times 1.84 \times 1.84 ~\upmu \hbox {m}^3$ for EPDM and PU. The number of projection was 1202 with an exposure time of 5 s per projection. The images were reconstructed with standard filtered backprojection.

## Data Availability

The data sets generated and/or analysed during the current study are available from the corresponding author on reasonable request.
